# Supplementary material for: Single-Cell Sequencing of Lung Macrophages and Monocytes Reveals Novel Therapeutic Targets in COPD
Source: Cells. 2023 Dec 5;12(24):2771. doi: 10.3390/cells12242771 (PMC10741950; doi:10.3390/cells12242771)
Supplement: Supplementary file 1 [file cells-12-02771-s001.zip › Fig.S1_Biological Functions - Cluster 2- supplement.pdf]

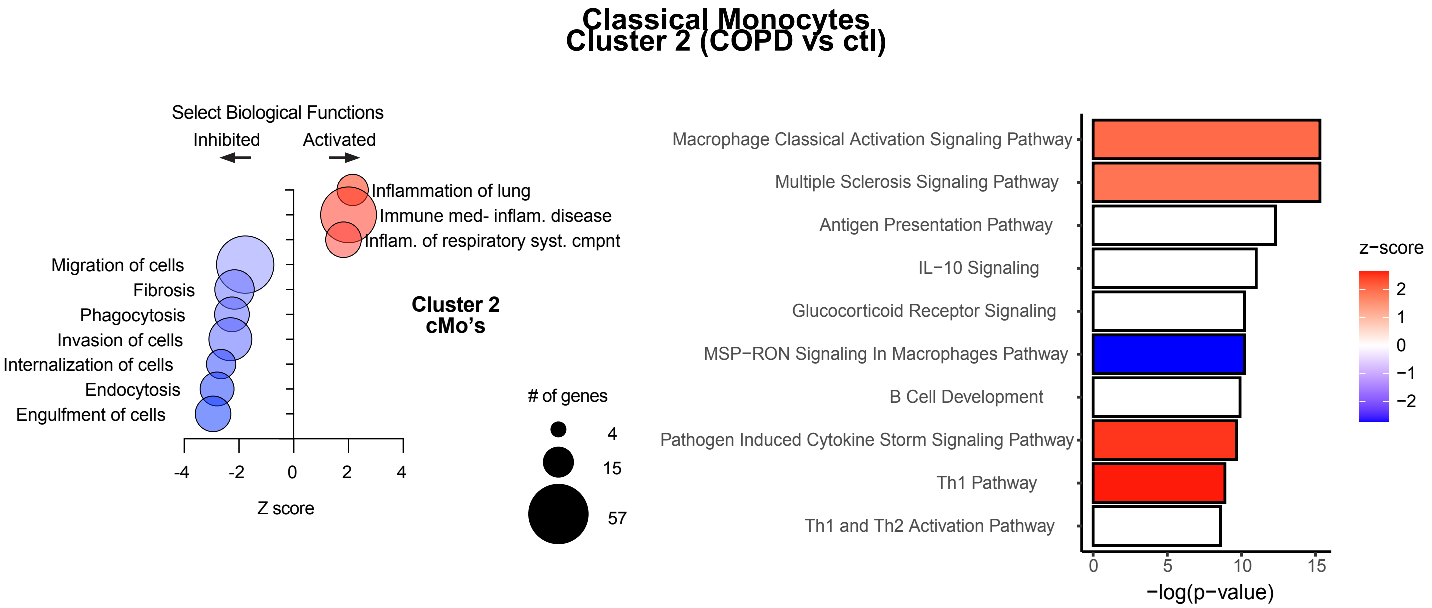

Supplementary Fig. 1. Top predicted downstream biological functions (A) and canonical pathways
(B) using Ingenuity Pathway Analysis of differentially expressed genes in COPD vs control cells from mixed classical monocyte cluster 2 (n = 160, Padj<0.05, $|log_2FC|>0.5$).
