# Supplementary material for: Single-Cell Sequencing of Lung Macrophages and Monocytes Reveals Novel Therapeutic Targets in COPD
Source: Cells. 2023 Dec 5;12(24):2771. doi: 10.3390/cells12242771 (PMC10741950; doi:10.3390/cells12242771)
Supplement: Supplementary file 1 [file cells-12-02771-s001.zip › Fig.S2_11.9.22.IPA Fluticasone - supplement.pdf]

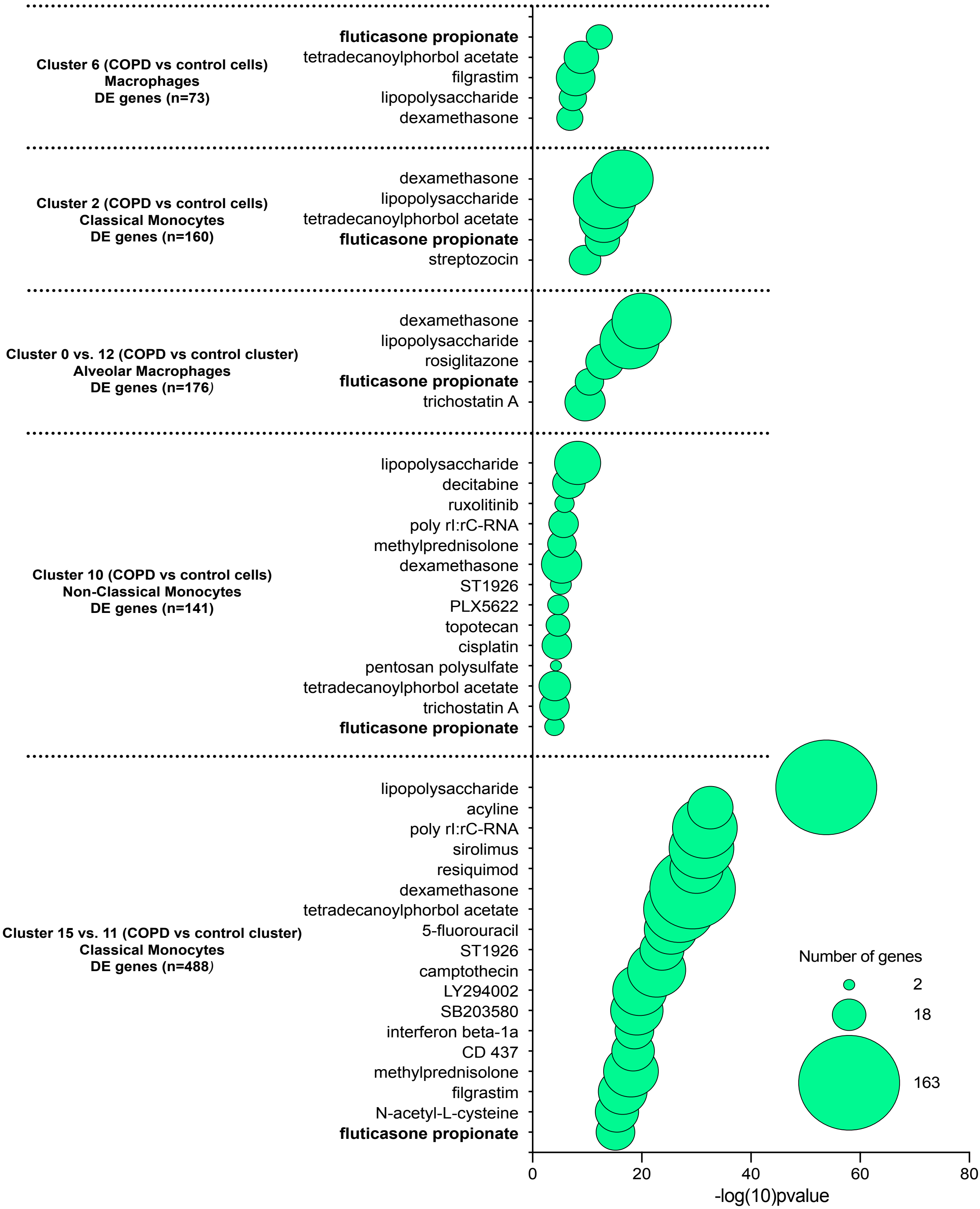

Supplementary Fig. 2. Top Biological and chemical drug targets for differentially expressed genes in mixed (COPD+control) and COPD-predominant clusters as predicted by Ingenuity Pathway Analysis.
