# Supplementary material for: Single-Cell Sequencing of Lung Macrophages and Monocytes Reveals Novel Therapeutic Targets in COPD
Source: Cells. 2023 Dec 5;12(24):2771. doi: 10.3390/cells12242771 (PMC10741950; doi:10.3390/cells12242771)
Supplement: Supplementary file 1 [file cells-12-02771-s001.zip › Fig.S3_Volcano_plots.pdf]

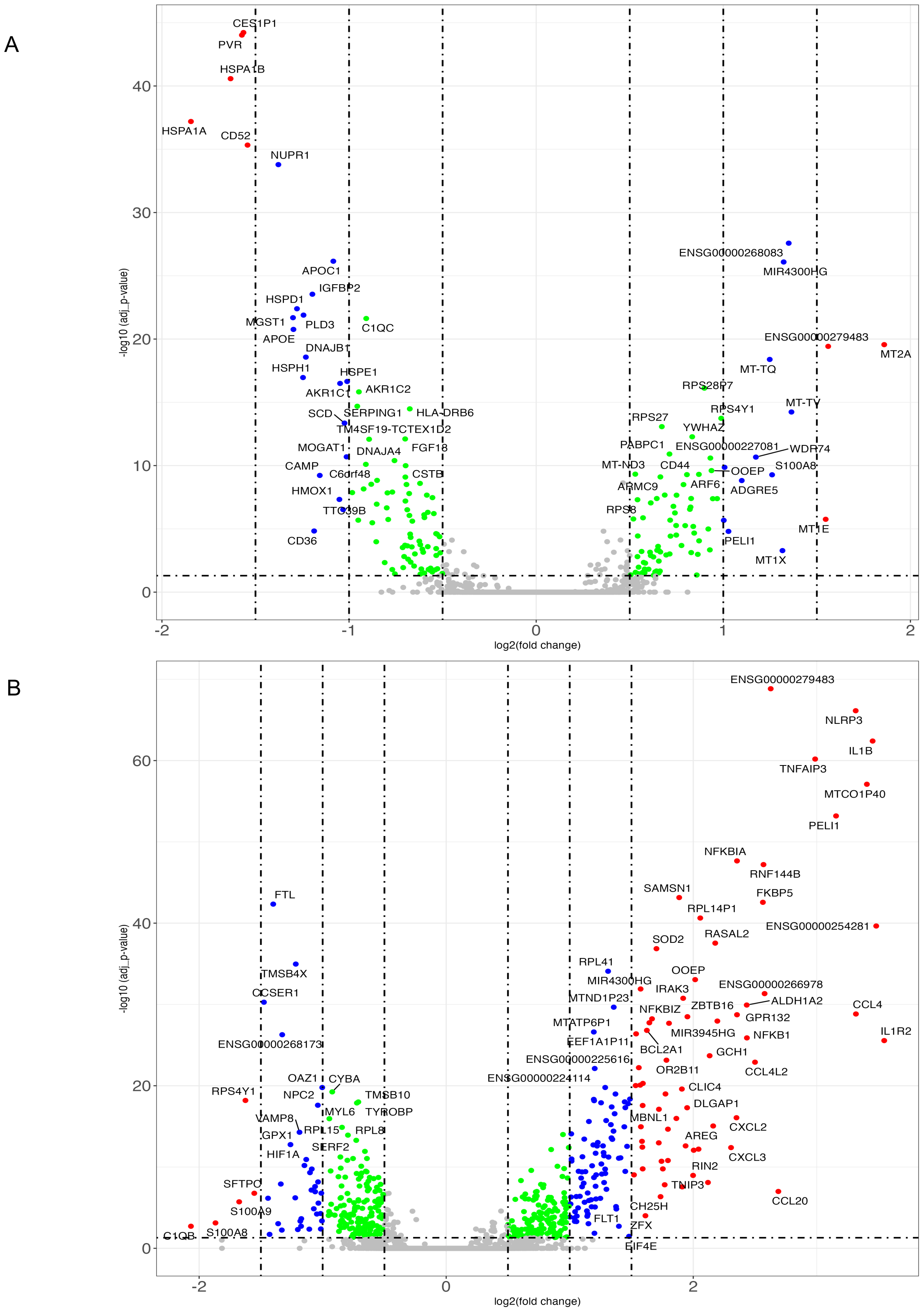

Fig.S3. Volcano plots of DE analysis. Grey dots represent insignificant genes that either has adjusted p-values larger than 0.05 or |log2FC|<0.5. For genes with significant adjusted p-values (less than 0.05), we use three colors to represent their magnitude of change. Red denotes the gene with |log2FC|>1.5, Blue stands for the gene which 1<|log2FC|<=1.5. Green represents for the gene with 0.5<|log2FC|<=1. (A) The DE analysis result of cluster 0 versus cluster 12. (B) The DE analysis result of cluster 15 versus cluster 11.
