# Supplementary material for: Single-Cell Sequencing of Lung Macrophages and Monocytes Reveals Novel Therapeutic Targets in COPD
Source: Cells. 2023 Dec 5;12(24):2771. doi: 10.3390/cells12242771 (PMC10741950; doi:10.3390/cells12242771)
Supplement: Supplementary file 1 [file cells-12-02771-s001.zip › Fig.S4_ViolinPlots_for significant genes.pdf]

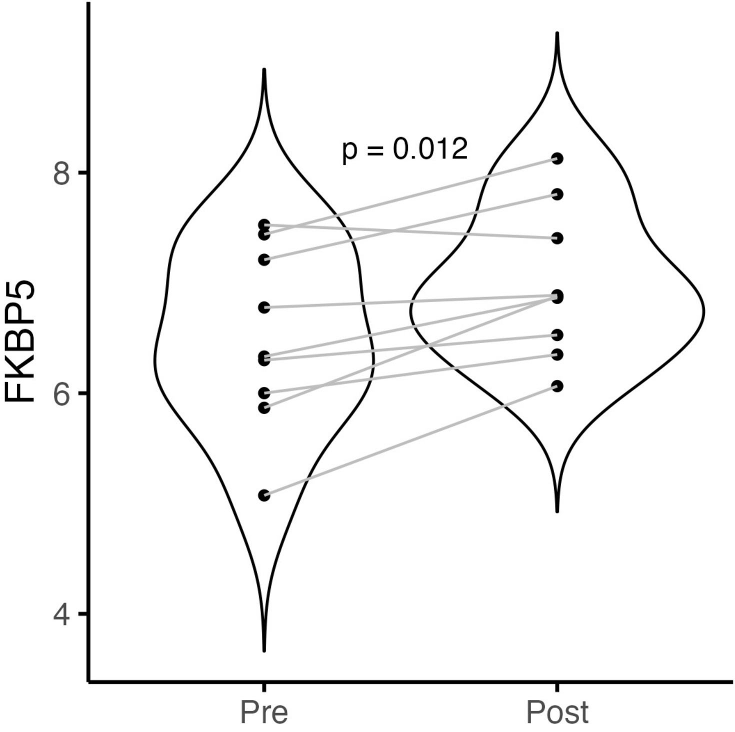
p = 0.012
FKBP5
8
6
4
Pre
Post

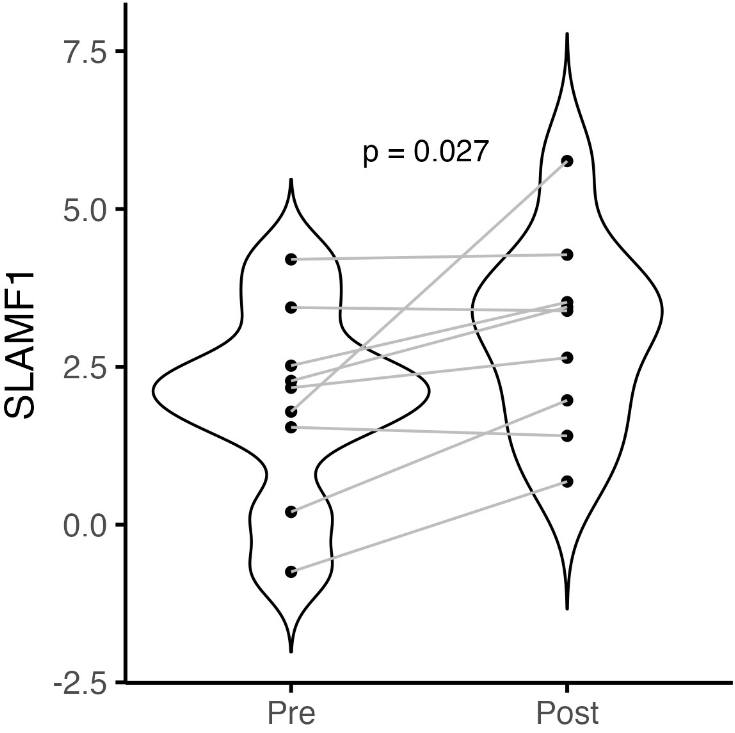
SLAMF1
7.5
5.0
2.5
0.0
-2.5
p = 0.027
Pre
Post

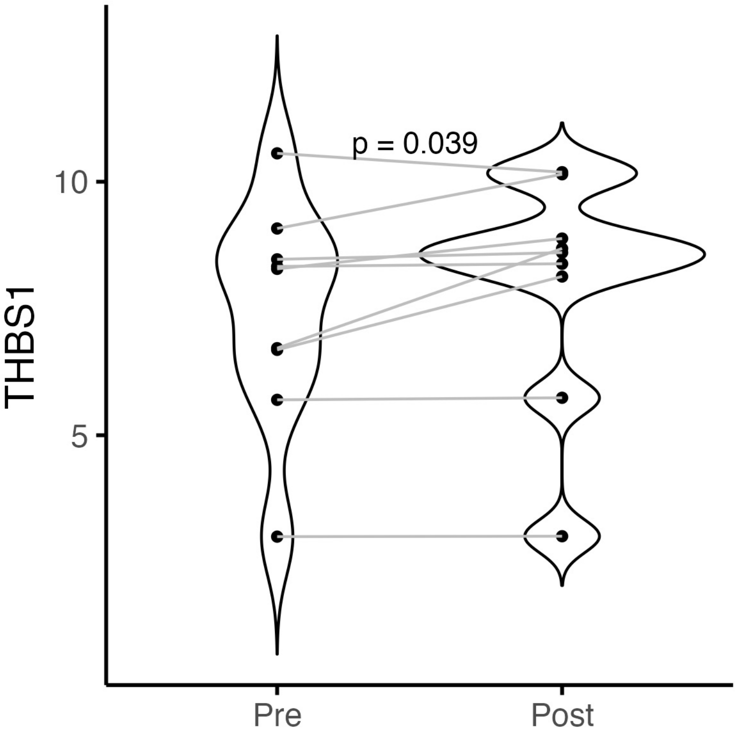

p = 0.039
10
5
THBS1
Pre
Post

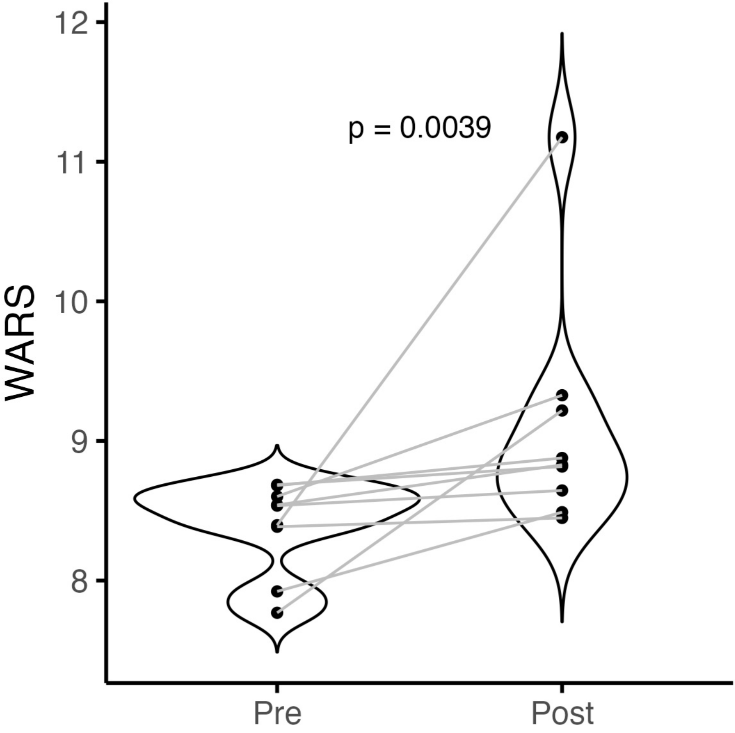

p = 0.0039
WARS
12
11
10
9
8
Pre
Post

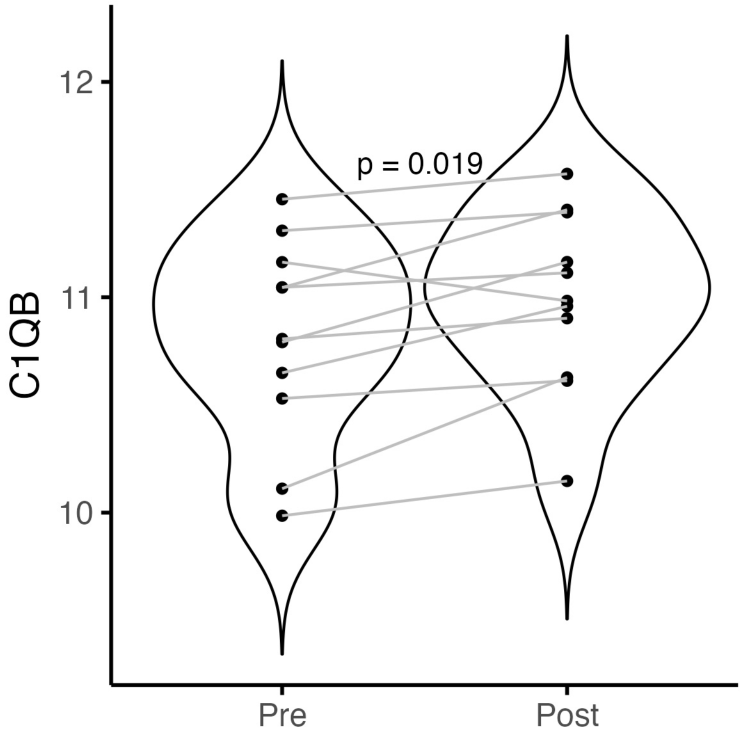

C1QB
12
11
10
p = 0.019
Pre
Post

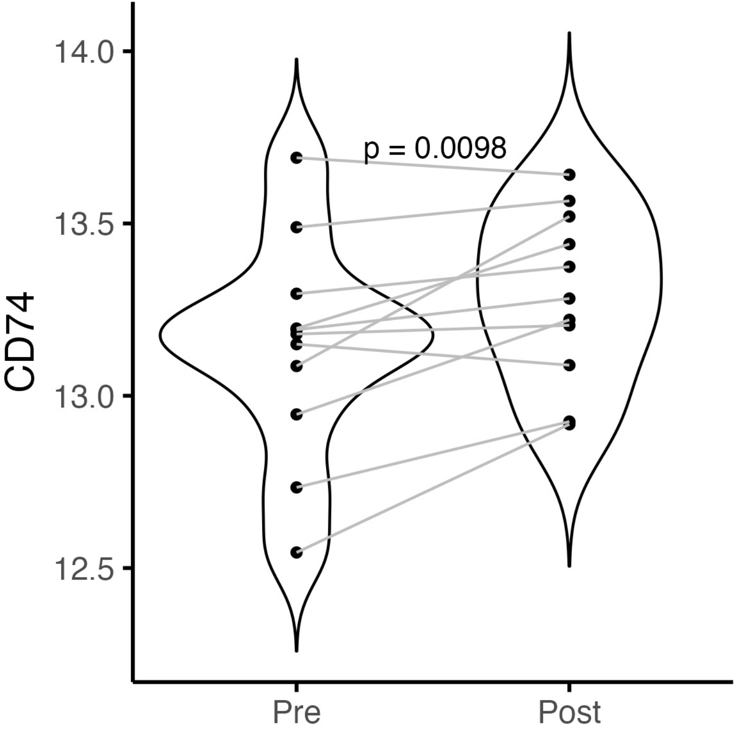

CD74
14.0
13.5
13.0
12.5
p = 0.0098
Pre
Post

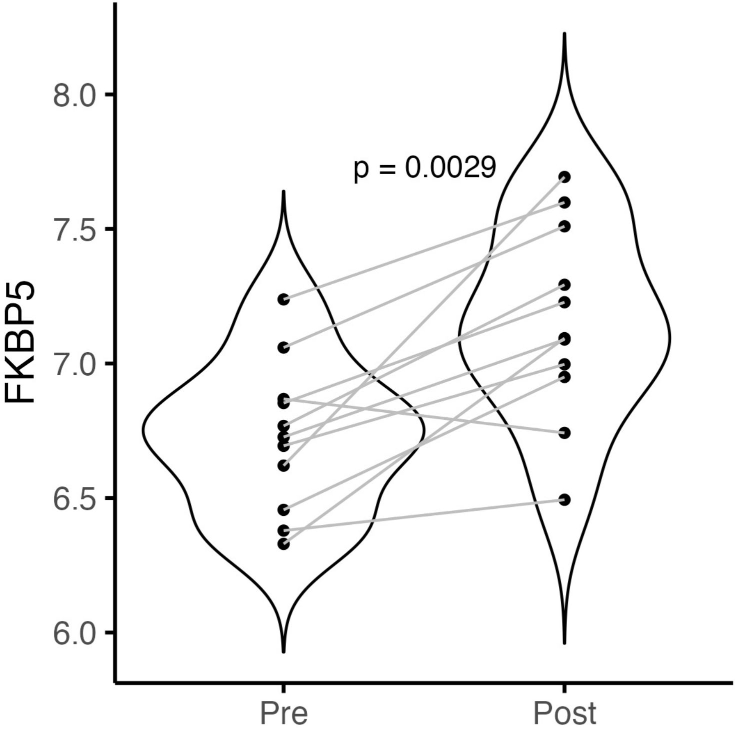
p = 0.0029
FKBP5
8.0
7.5
7.0
6.5
6.0
Pre
Post

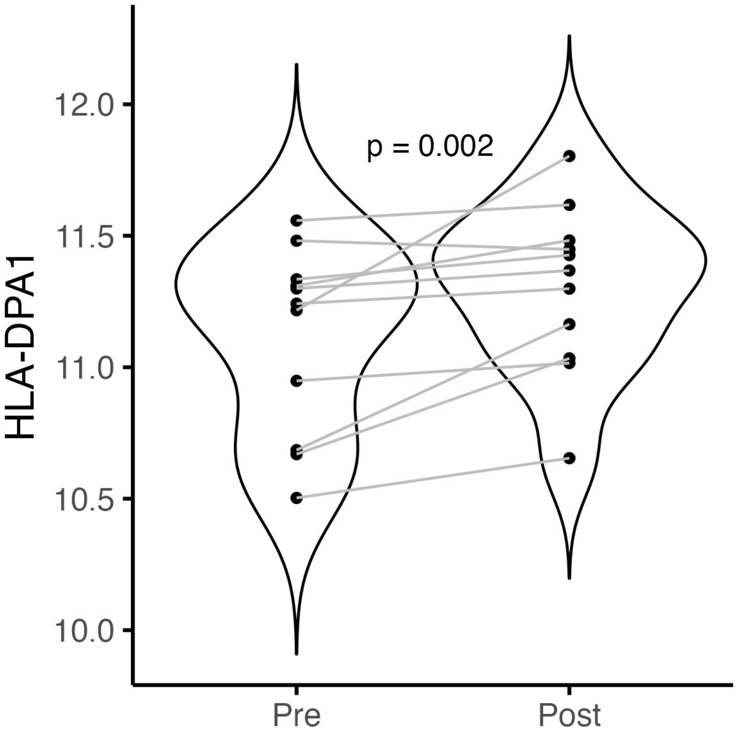

HLA-DPA1
12.0
11.5
11.0
10.5
10.0
p = 0.002
Pre
Post

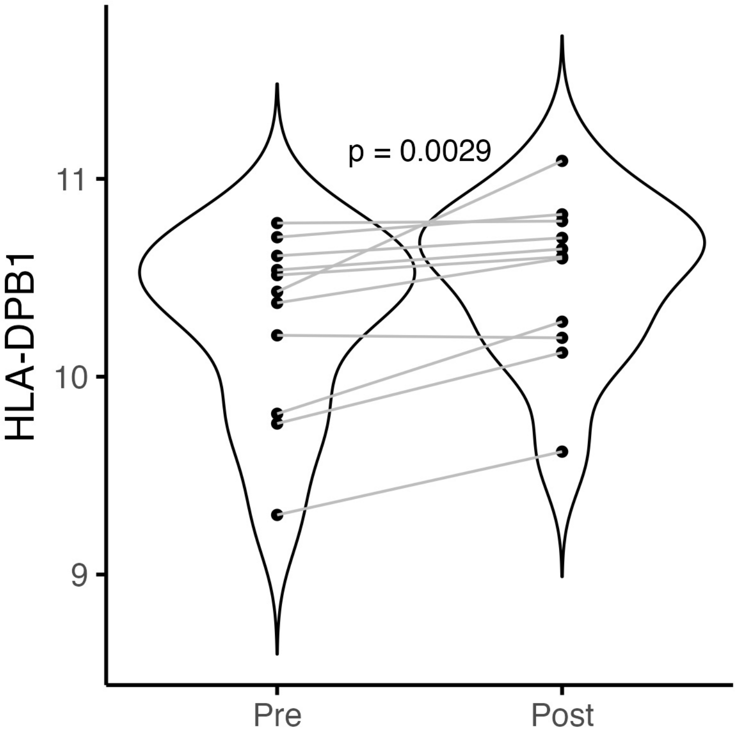
p = 0.0029
HLA-DPB1
11
10
9
Pre
Post

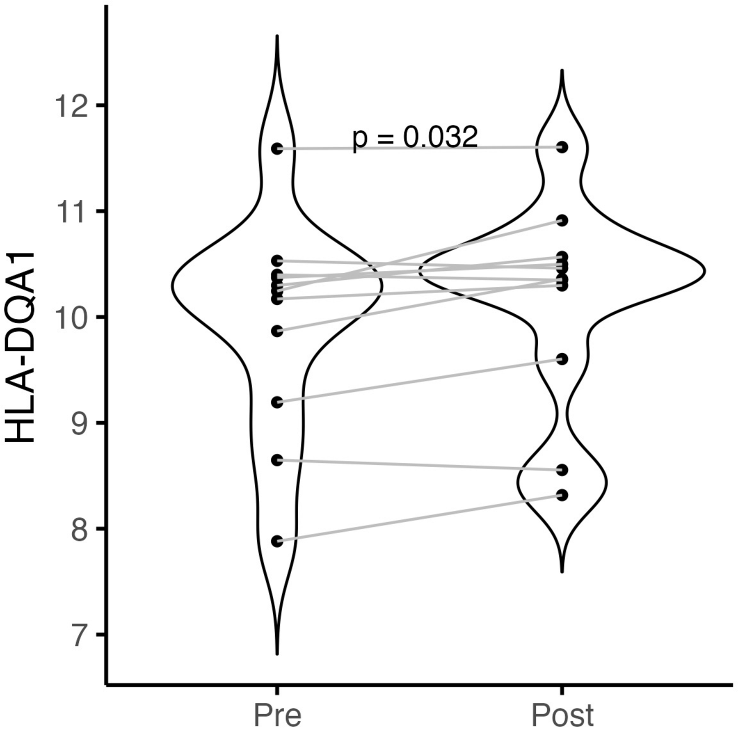
p = 0.032
HLA-DQA1
12
11
10
9
8
7
Pre
Post

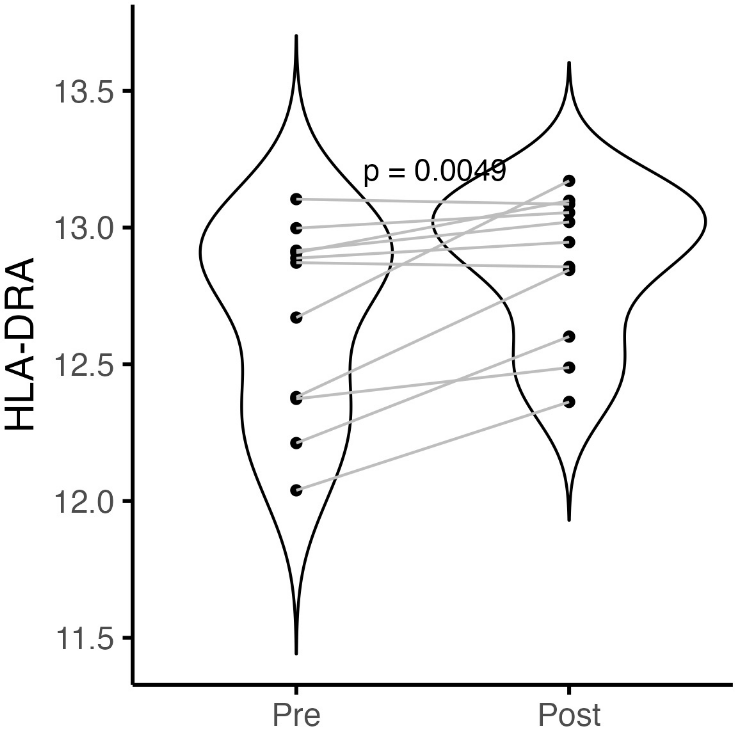

13.5
13.0
12.5
12.0
11.5
HLA-DRA
p = 0.0049
Pre
Post

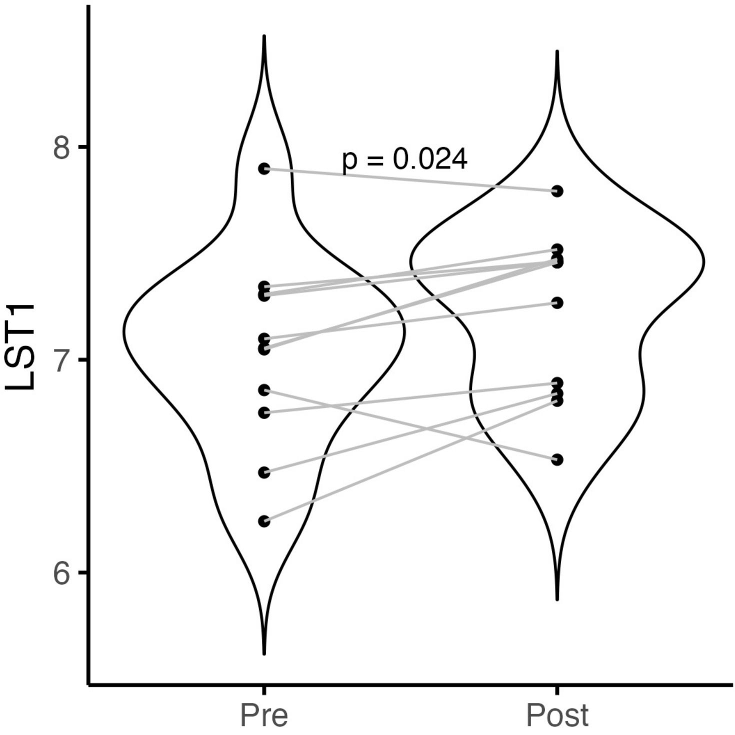
8
7
6
LST1
p = 0.024
Pre
Post

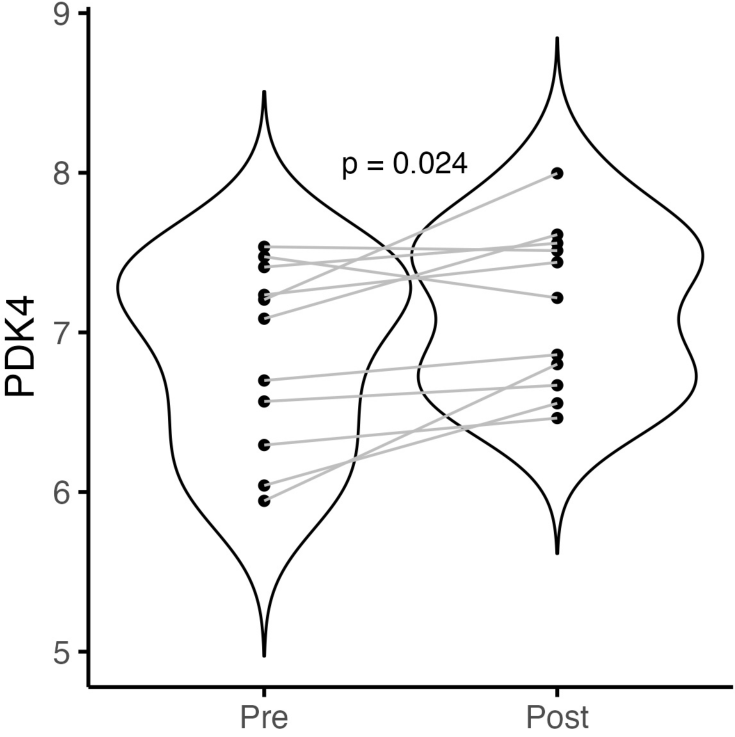

9
8
7
6
5
PDK4
p = 0.024
Pre
Post
