# Supplementary figures and images for: Single-Cell Sequencing of Lung Macrophages and Monocytes Reveals Novel Therapeutic Targets in COPD
Source: Cells. 2023 Dec 5;12(24):2771. doi: 10.3390/cells12242771 (PMC10741950; doi:10.3390/cells12242771)

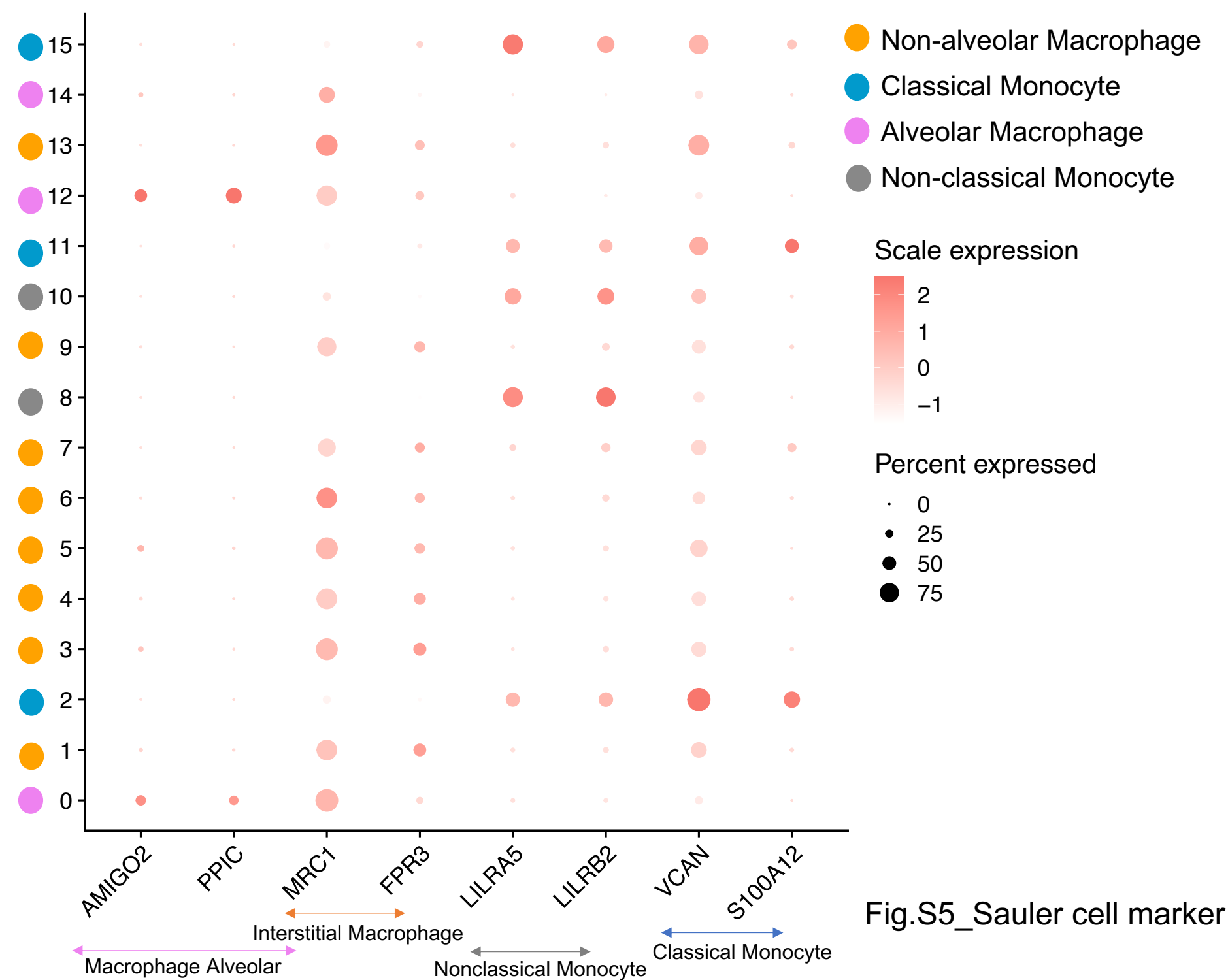

Fig.S5\_Sauler cell marker

Supplement: Supplementary file 1 [file cells-12-02771-s001.zip › Fig.S5_Sauler cell marker.pdf]

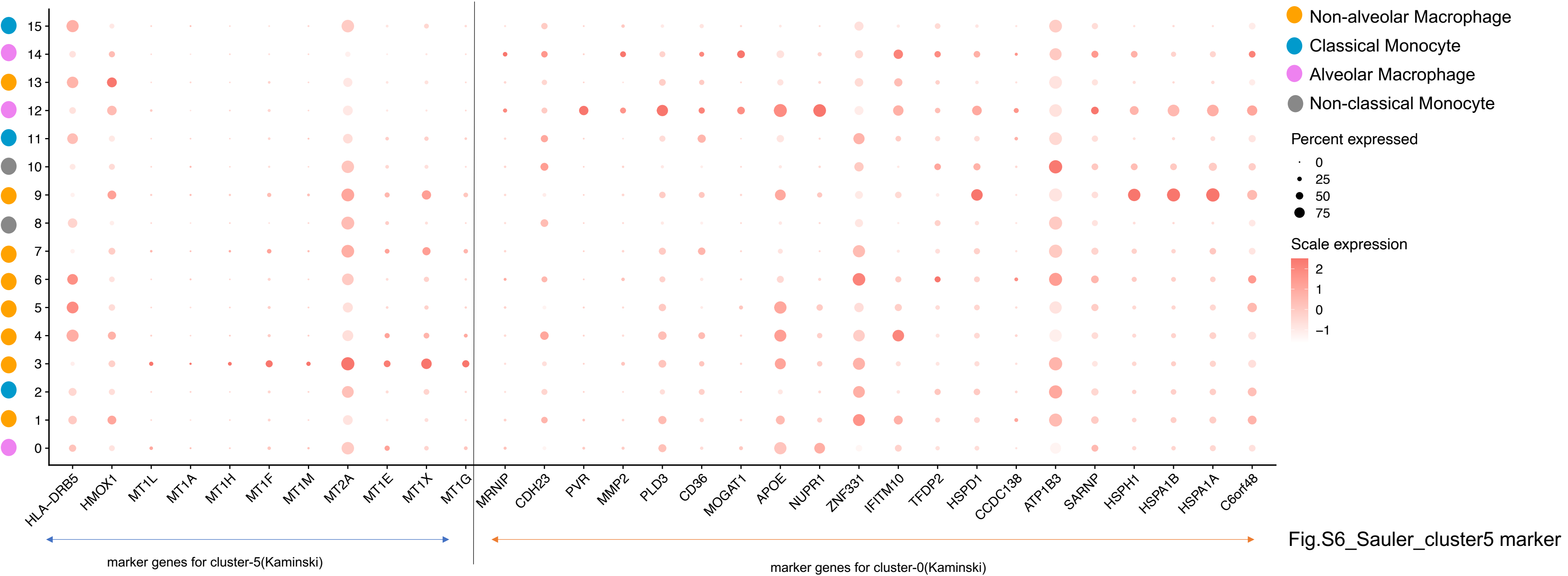

Supplement: Supplementary file 1 [file cells-12-02771-s001.zip › Fig.S6_Sauler_cluster5 marker.pdf]

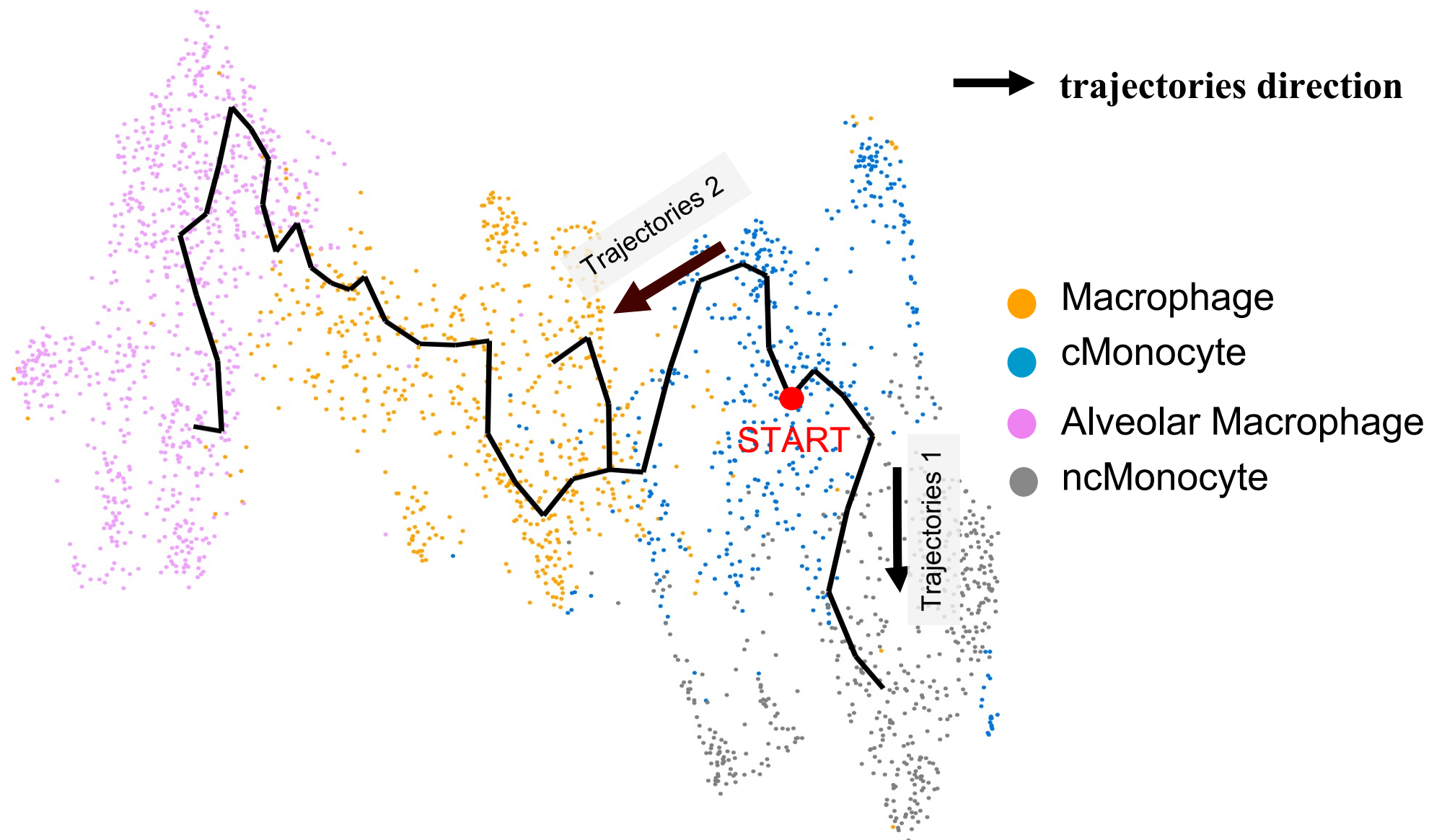

Fig.S7\_ pseudo-time analysis only on COPD

Supplement: Supplementary file 1 [file cells-12-02771-s001.zip › Fig.S7_ pseudo-time analysis only on COPD .pdf]

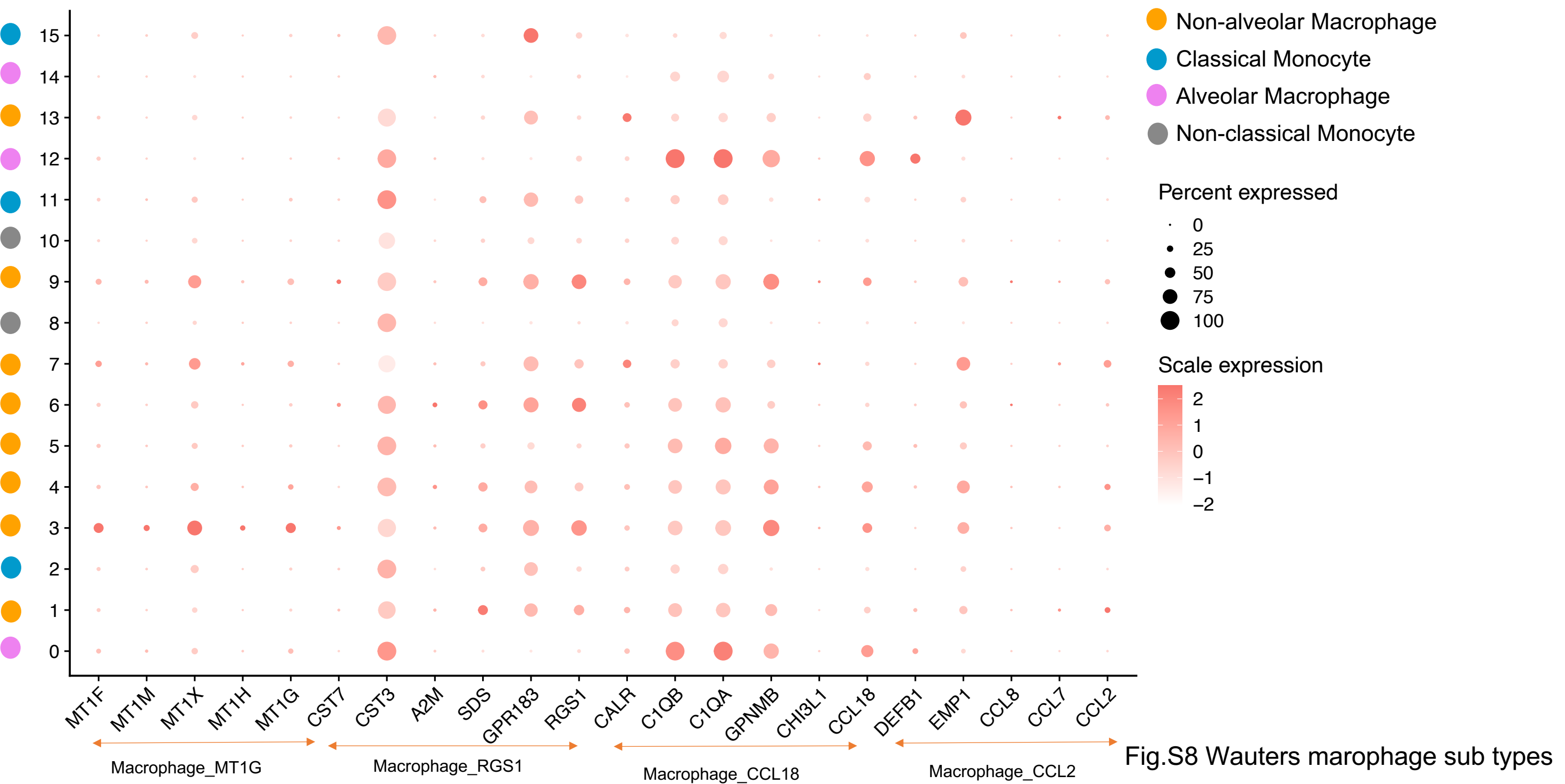

Supplement: Supplementary file 1 [file cells-12-02771-s001.zip › Fig.S8 Wauters marophage sub types.pdf]

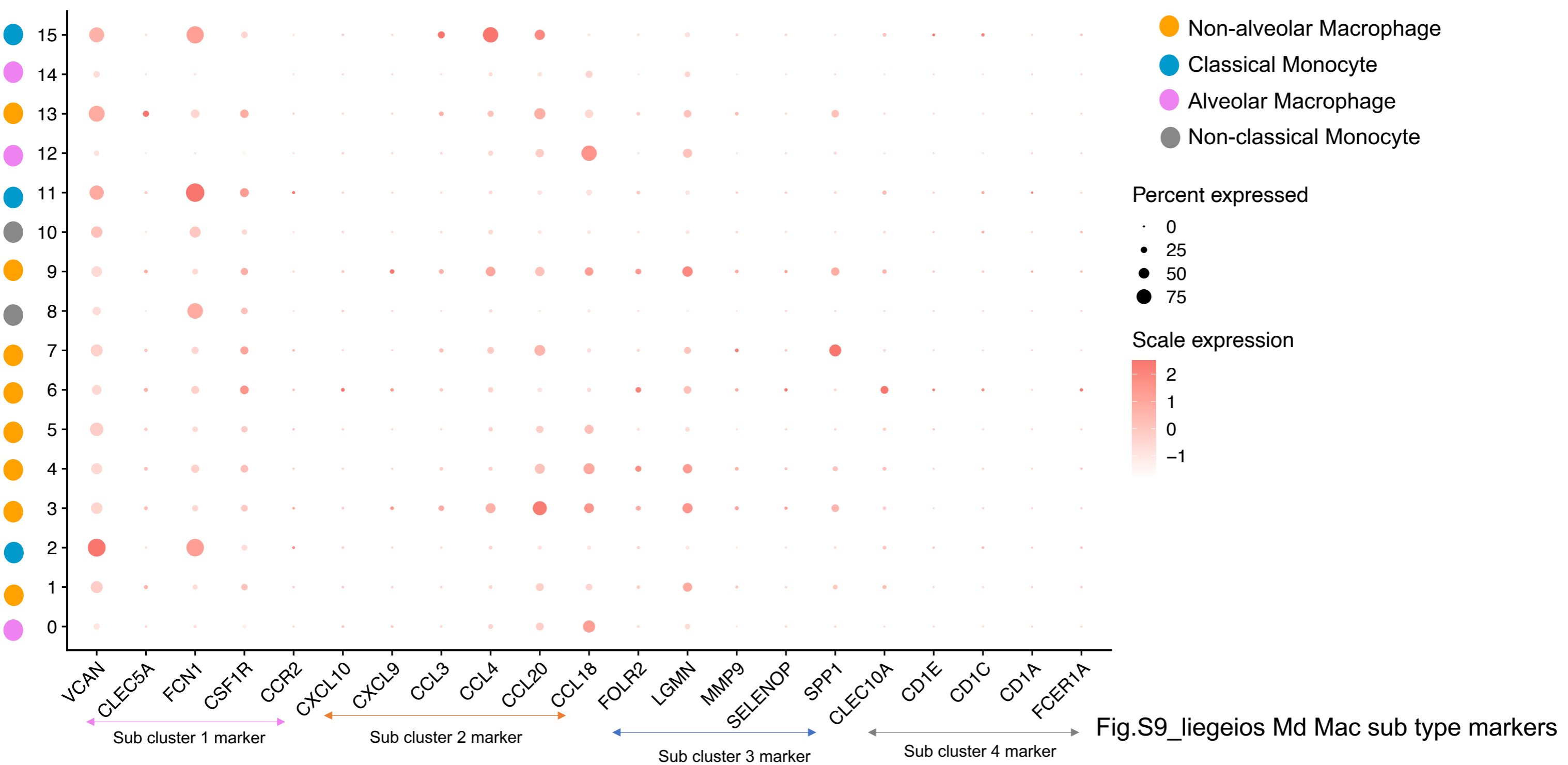

Supplement: Supplementary file 1 [file cells-12-02771-s001.zip › Fig.S9_liegeios Md Mac sub type markers.pdf]
